# Social network-based measurement of abortion incidence: promising findings from population-based surveys in Nigeria, Cote d’Ivoire, and Rajasthan, India

**DOI:** 10.1186/s12963-020-00235-y

**Published:** 2020-10-19

**Authors:** Suzanne O. Bell, Mridula Shankar, Elizabeth Omoluabi, Anoop Khanna, Hyacinthe Kouakou Andoh, Funmilola OlaOlorun, Danish Ahmad, Georges Guiella, Saifuddin Ahmed, Caroline Moreau

**Affiliations:** 1 Department of Population, Family and Reproductive Health, Johns Hopkins Bloomberg School of Public Health, Baltimore, MD USA; 2 Department of Statistics and Population Studies, University of the Western Cape, Cape Town, South Africa; 3 Centre for Research, Evaluation Resources and Development, Ile-Ife, Nigeria; 4 Indian Institute of Health Management Research, Jaipur, India; 5 Programme National de Santé de la Mère et de l’Enfant (PNSME), Abidjan, République de Côte d’Ivoire; 6 College of Medicine, University of Ibadan, Ibadan, Nigeria; 7 Institut Supérieur des Sciences de la Population (ISSP), University of Ouagadougou, Ouagadougou, Burkina Faso; 8 Soins Primaires et Prévention, CESP Centre for Research in Epidemiology and Population Health, U1018, Inserm, F-94800 Villejuif, France

**Keywords:** Abortion, Measurement, Survey

## Abstract

**Background:**

Monitoring abortion rates is highly relevant for demographic and public health considerations, yet its reliable estimation is fraught with uncertainty due to lack of complete national health facility service statistics and bias in self-reported survey data. In this study, we aim to test the confidante methodology for estimating abortion incidence rates in Nigeria, Cote d’Ivoire, and Rajasthan, India, and develop methods to adjust for violations of assumptions.

**Methods:**

In population-based surveys in each setting, female respondents of reproductive age reported separately on their two closest confidantes’ experience with abortion, in addition to reporting about their own experiences. We used descriptive analyses and design-based *F* tests to test for violations of method assumptions. Using post hoc analytical techniques, we corrected for biases in the confidante sample to improve the validity and precision of the abortion incidence estimates produced from these data.

**Results:**

Results indicate incomplete transmission of confidante abortion knowledge, a biased confidante sample, but reduced social desirability bias when reporting on confidantes' abortion incidences once adjust for assumption violations. The extent to which the assumptions were met differed across the three contexts. The respondent 1-year pregnancy removal rate was 18.7 (95% confidence interval (CI) 14.9–22.5) abortions per 1000 women of reproductive age in Nigeria, 18.8 (95% CI 11.8–25.8) in Cote d’Ivoire, and 7.0 (95% CI 4.6–9.5) in India. The 1-year adjusted abortion incidence rates for the first confidantes were 35.1 (95% CI 31.1–39.1) in Nigeria, 31.5 (95% CI 24.8–38.1) in Cote d’Ivoire, and 15.2 (95% CI 6.1–24.4) in Rajasthan, India. Confidante two’s rates were closer to confidante one incidences than respondent incidences. The adjusted confidante one and two incidence estimates were significantly higher than respondent incidences in all three countries.

**Conclusions:**

Findings suggest that the confidante approach may present an opportunity to address some abortion-related data deficiencies but require modeling approaches to correct for biases due to violations of social network-based method assumptions. The performance of these methodologies varied based on geographical and social context, indicating that performance may be better in settings where abortion is legally and socially restricted.

## Background

Regardless of legality, induced abortion is practiced throughout the world. Recent estimates suggest a global annual abortion incidence of 35 abortions per 1000 women age 15 to 44, ranging from 17 in North America to 44 in Latin America [1]. While monitoring abortion rates is highly relevant for demographic and public health considerations, its estimation is fraught with uncertainty due to lack of accurate or complete reporting in national service statistics and bias in self-reported survey data.

With regard to health facility service statistics, there are two primary challenges. In low-resource settings, providers often fail to record postabortion care (PAC) and abortion services to national health registries. Although this issue is not unique to abortion, it is exacerbated by the sensitive nature and legal status of pregnancy termination. Moreover, self-induced abortions (using misoprostol with or without mifepristone, or other drugs or methods) and abortions performed by providers outside the formal health care system are not captured through service statistics if these women did not subsequently seek PAC in a health facility.

To address these limitations, researchers have long relied upon statistical techniques that adjust health facility service statistics or conducted community-based surveys for producing more accurate estimates of abortion in low- and middle-income country settings. The Guttmacher Institute developed the Abortion Incidence Complications Methodology (AICM) in the 1990s and has refined and adapted the method for different contexts [2]. This methodology includes a health facility survey that generates a nationally representative estimate of the number of women receiving PAC, and if legal, abortion. Investigators also survey key informants to produce a set of adjustment factors, which they use with the facility service statistics to account for the abortions occurring outside of the formal health care system. With the increasing availability of medication abortion drugs, this standard AICM methodology is being challenged, and researchers are making further modifications [3–7]. Additionally, while the AICM allows for estimation of overall levels of abortion, assessing detailed characteristics of women having abortions—other than those presenting for PAC—and the safety of the abortions specific types of women receive is not possible. These challenges limit the ability to identify and serve the most vulnerable and at-risk populations with optimal public health interventions.

Community-based surveys on abortion allow for collection of individual women’s characteristics, but there is significant concern regarding the validity of respondent abortion reporting. Direct questioning in face-to-face surveys results in substantial underreporting of abortion, even in settings where abortion is legal [8, 9]. Underreporting varies by women’s sociodemographic characteristics, which prevents simple calibration of survey estimates [9, 10]. While audio computer-assisted self-interview (ACASI) has the potential to reduce the social desirability pressure of reporting a sensitive behavior, it has not consistently outperformed direct questioning [11–13]. Moreover, applying ACASI in low-literacy or low-computer/smartphone literacy populations is challenging.

An alternative approach is to ask about sensitive items indirectly in order to reduce the impact of social desirability. Specifically related to induced abortion, researchers have employed indirect techniques such as the randomized response technique (RRT) and the list experiment to indirectly ask respondents about their own experience with abortion. However, the performance of these methods in comparison to direct self-reports has been mixed [14–21]. Another group of indirect methodologies relies on multiplicity sampling: asking respondents to report on the experiences of multiple people in their social network [22, 23]. The anonymous third-party reporting (ATPR) method is an adaptation of social network-based approaches that investigators have used specifically to measure abortion [24, 25]. The method consists of respondents identifying their entire social network of reproductive age women, specifically women “who shared or could have shared intimate information with the respondent over the past year” [24], and for each identified woman, asking if she had an abortion in each of the 5 years prior to the survey. The method proved effective in Burkina Faso [24] but did not result in more valid estimates than self-report in Rajasthan, suggesting its utility may be context dependent [24, 25]. A simplified version of the ATPR, asking only about one best friend’s experience, seemed to outperform direct questioning in both Malawi and Texas [26, 27].

Building on these social network strategies, researchers at the Guttmacher Institute have suggested collecting information on a small, fixed number of respondents’ closest confidantes and their experiences with abortion [28]. This adaptation, referred to as the confidante methodology, incorporates the relationship description from the ATPR, which emphasizes sharing of sensitive information, and the fixed number of friends from the best friend methodology. The ATPR relationship description may ensure respondents report only on women in their social network with whom the sharing of personal information (like abortion) would occur, while the small, fixed number of confidantes may reduce the likelihood of underestimating the sensitive behavior by excluding women with whom the respondent is less close. This indirect strategy allows researchers to collect sociodemographic characteristics and abortion experience details of respondents’ confidante(s), which other indirect methods had not explored.

Social network-based methodologies, such as the confidante method, are based on several sociological assumptions that need to be met in order to avoid bias. The assumptions include that: (1) respondents know about the sensitive behaviors of their confidantes (i.e., that there is no transmission bias whereby information on the behavior of interest is not “transmitted” from a confidante to a respondent); (2) the confidante sample characteristics resemble that of the respondent sample, thus providing a surrogate, representative sample of the population of interest; and (3) social desirability pressure is reduced when reporting on the stigmatized behaviors of one’s confidantes as opposed to oneself [29–33]. However, the validity of these assumptions and their implications on abortion estimations are not known. Using more advanced analytic techniques that correct for biases in the confidante sample could further improve the validity and precision of the abortion incidence estimates produced from the confidante data.

In this study, we aim to test the confidante methodology for estimating abortion incidence rates in Nigeria, Cote d’Ivoire, and Rajasthan, India, and develop methods to adjust for violations of assumptions [24, 27, 28]. Specifically, we test the three assumptions of this social network-based indirect methodology by: (1) examining the presence of transmission bias; (2) assessing the confidante sample representativeness, and; (3) examining if the confidante methodology reduces social desirability bias. We then use advanced analytic techniques that correct for violations of these assumptions in order to improve the validity and precision of the abortion incidence estimates produced from the confidante data.

The three sites were selected for a number of reasons: First, the legality of abortion and the availability of safe abortion services differ substantially across these countries (legal in India and highly restricted in Nigeria and Cote d’Ivoire); second, prior indirect methodologies seemed to have worked differently in West Africa compared to India [24, 25], indicating that context is important to method performance; third, there is a data gap in abortion-related estimates in West African countries, including the incidence of abortion and the proportion that are unsafe; and finally, these sites are part of a larger project conducting frequent population-based surveys of reproductive age women, permitting testing of this methodology in samples representative at the state or national levels [34, 35]. The findings from this study will allow us to evaluate and compare the utility of this parsimonious indirect approach in producing more valid abortion incidence estimates in different contexts.

## Methods

### Data

Data for this study come from the Performance Monitoring and Accountability 2020 (PMA2020) surveys in Nigeria, Cote d’Ivoire, and Rajasthan, India. PMA2020 conducts population-based surveys of reproductive age women (15 to 49 years old) using a multi-stage stratified cluster sampling design with probability proportional to size cluster sampling to produce nationally or state representative household and female samples. These surveys are implemented annually with rapid turnaround of results in order to track key family planning and reproductive health indicators. The sampling methodology has been described in detail previously [34, 35].

Trained female resident interviewers conducted face-to-face interviews with all consenting women aged 15 to 49 residing in sampled households. In the most recent survey rounds in each location (round 5 in Nigeria, round 2 in Cote d’Ivoire, and round 4 in Rajasthan), researchers added a module on abortion to the core female questionnaire. Data collection occurred from April through May 2018 in Nigeria, from July through August 2018 in Cote d’Ivoire, and from April through June 2018 in Rajasthan. Interviewers conducted surveys using the English questionnaire or the translated versions in Hausa, Igbo, Yoruba, and Pidgin in Nigeria; French in Cote d’Ivoire; and Hindi in Rajasthan. Interviewers could also conduct interviews using local dialects to improve respondent comprehension, which they would translate orally. These oral translations were first agreed upon in language groups during trainings. The Johns Hopkins Bloomberg School of Public Health provided ethical approval for this study (8308), as did the National Health Research Ethics Committee of Nigeria (NHREC/01/01/2007-02/01/2018C), the Indian Institute of Health Management Research (IIHMR) Institutional Review Board for Protection of Human Subjects in Rajasthan (Feb 2018 1), and the Comite National D’ Etique de la Recherche (CNER) in Cote d’Ivoire (N/Ref: 036-18/MSHP/CNER-kp).

### Measurement

The newly added module collected abortion data using two data collection techniques to generate estimates of abortion incidence at the national and/or state levels. Prior to any suggestive mention of abortion, interviewers obtained information on up to two of the respondent’s closest confidantes. We chose two confidantes to test whether confidante selection bias and abortion information transmission deteriorates between the closest and second closest confidante while not expanding the questionnaire substantially by including third or higher order confidantes. Following prior applications of the ATPR method, we defined confidantes as female friends or relatives age 15 to 49 living in the country “whom you share very personal information with and who also share their very personal information with you” [24].

Interviewers first asked respondents for the number of female friends or relatives between the ages of 15 and 49 living in the country whom they considered “confidantes” using the aforementioned definition. If the respondent reported more than one confidante, the interviewer asked her to identify her “closest” female friend or relative first. For confidante two, the interviewer asked the respondent to identify her “next closest” female friend or relative. For each confidante, we had the respondent provide a fake name in order to easily refer to the woman in later questions and collected information on the confidante’s age and level of education. We collected information on each confidante prior to introducing the abortion module to minimize respondents preferentially selecting confidantes who had previously undergone abortions, which could bias the confidante abortion incidence upwards.

Next, for each of the two confidantes, interviewers asked a question on their experiences with pregnancy removal when they were pregnant or worried they could be. We used this terminology in lieu of asking a question using the direct translation of “abortion” as this is a more descriptive and less stigmatizing way to refer to this event. This language was validated during the piloting and formative assessment of the surveys in each setting, using analogous phrasing in each local language for the translated instruments that interviewers used during fieldwork. The prelude to this section framed the questions in terms of actions women may take when they become pregnant at a time when they cannot or do not want to be pregnant in order to minimize reporting of miscarriage. For each reported pregnancy removal, we obtained information on the year it most recently occurred, the first and last or only method(s) used, provider(s) or source(s) of these method(s), and whether the confidante visited a health facility for treatment of (perceived) complications in the process of terminating the pregnancy. Subsequently, we asked similar questions on the respondent’s own experiences with pregnancy removal. In the remainder of this article, we use the term abortion to refer to pregnancy removal. We focus our analysis on the comparison of direct (respondent) versus indirect (confidante) reporting of abortion.

### Analyses

We present the analytic methods specific to testing each of the three social network-based indirect methodology assumptions and then discuss the approach we used to adjust our indirect estimates of abortion incidence to account for potential bias arising from the violation of these assumptions.

### Assumption 1: transmission of abortion knowledge

In order to evaluate whether abortion knowledge is fully transmitted from confidantes to respondents (i.e., whether there is transmission bias), we first evaluated whether respondents all had confidantes, a pre-condition to sharing. To do so, we first tested for differences in the socioeconomic and reproductive characteristics of respondents by number of reported confidantes (zero, one or more, and two or more). We then assessed whether respondents who reported their own abortion indicated they told each confidante; we assumed the level of respondent sharing with their confidantes mirrored sharing in the other direction. We used design-based *F* tests to assess statistical significance.

### Assumption 2: confidante sample representativeness

To use the confidante data to calculate population-level estimates of annual abortion rates, we must assume that the surrogate sample created by the confidante data is representative of the population of reproductive age women. The “missing” confidantes who correspond to respondents who reported zero confidantes may contribute to selection bias in the confidante sample. In addition, respondents may describe confidantes that, on average, have different characteristics than themselves, further contributing to confidante sample distortions. Since the respondent sample—when weighted to account for the complex sampling design, probability of selection, and non-response—*is* representative of women of reproductive age, we compared the distribution of age and education (the two sociodemographic indicators available for confidantes) between the respondent sample and confidantes one and two samples, separately in each site. We used design-based *F* tests to assess whether differences were statistically significant.

### Assumption 3: reduced social desirability pressure when reporting on confidante as opposed to self

To assess whether reporting on a confidantes’ sensitive behavior reduces social desirability pressure, we calculated separate 1-year incidence rates of abortion for each sample, i.e., respondent, confidante one, and confidante two. Next, we tested whether confidantes one and two abortion incidence rates were statistically significantly different from the respondent rates using Poisson regression with the independent variable being a dichotomous indicator for respondent versus confidante one or two (assessed using separate models for each confidante).

We calculated 1-year abortion incidence rates by determining the number of abortions reported in 2017 and in 2018 divided by the number of women in each sample. To convert the proportion into a 1-year incidence rate, we divided the estimate by the total number of years covered from January 1, 2017, through the date of the interview in 2018. We then multiplied the value by 1000 to generate the 1-year abortion rate per 1000 women age 15 to 49. We scaled the standard errors in the same manner. We weighted the incidence estimates and adjusted variances for respondent and confidante estimates to account for the complex survey sampling design and associated clustering.

For respondents who reported “don’t know” with regard to whether a confidante had ever had an abortion, we conservatively assumed that her confidante had not had one in the year prior to the survey for the purpose of incidence estimation. If a respondent reported a confidante likely had an abortion but was not completely certain, we excluded these cases in the unadjusted incidence estimation. Additionally, in Nigeria, we excluded respondent and confidante abortions that only involved the use of emergency contraception (EC) with no additional care as we suspect these were not in fact abortions. EC was not a separate method response option in other countries, thus any EC-only use would be included in “other pills”.

### Adjusting for violations of confidante method assumptions

In light of evidence that suggested transmission bias (Assumption 1 violation), we sought to adjust for it in two ways. First, we included confidante abortions that respondents reported with less certainty (response option “Yes, I think so”) but where the respondent could still report the method(s) used, in addition to those reported as definite (response option “Yes, I am certain”). Second, for respondents who reported no confidantes—or for those with only one confidante in the context of the confidante two estimates—their corresponding confidantes one and two data are essentially “missing”. In addition to potential transmission bias, this could result in selection bias with a non-representative confidante sample that violates Assumption 2. To address these biases, we ran separate Poisson models for each confidante sample using the respondent socioeconomic variables and the indicator variable for whether their corresponding confidante one (and confidante two in a separate model) had an abortion in the year prior as the outcome. We then used this model to predict the confidante probability of having an abortion in the prior year for “missing” observations in each of the surrogate confidante samples, that is, confidantes who were not in the sample because they had no close friends who we could have captured in the respondent sample. We combined this “imputed” information with respondent-reported confidante abortion data to estimate the probability of abortion in the prior year for confidante one and confidante two samples. This modeling approach is similar to mortality rate estimation work using survey data based on the sibling method where women with zero siblings are underrepresented [36]. Using the characteristics of respondents who reported having no confidantes (or zero or one confidante in the case of confidante two), we first adjusted confidante samples by including these respondents’ characteristics for the missing confidantes as we assume the missing confidantes on the whole are similar in characteristics to these respondents. As a final adjustment to the confidante data, we constructed post-stratification weights to ensure confidante characteristics matched respondents'.

We weighted all results and adjusted variances using the Taylor linearization approach to account for the complex sampling design and clustering. We conducted all analyses in Stata version 15.1 [37].

## Results

Interviewers completed surveys with 11,106 women in Nigeria; 2738 women in Cote d’Ivoire; and 5832 women in Rajasthan (Tables 1, 2, and 3). Response rates for the female survey were approximately 98% in all three countries.

**Table 1:** Characteristics of female respondents age 15 to 49 overall and by number of reported female confidantes in Nigeria

	All respondents	0 confidantes	≥ 1 confidante	≥ 2 confidantes
*N*	11,106	4788	5883	1953
Age				
15–19	18.9	17.5	**19.8**	18.8
20–24	16.2	14.9	**17.5**	16.5
25–29	18.8	17.2	**20.1**	19.0
30–34	15.0	15.0	**15.0**	15.5
35–39	13.9	15.5	**12.7**	14.0
40–44	10.5	11.7	**9.2**	10.0
45–49	6.8	8.2	**5.7**	6.2
Education				
Never	17.5	19.0	**14.8**	**15.9**
Primary	15.2	16.5	**14.2**	**12.1**
Secondary	46.9	46.9	**48.1**	**44.6**
Higher	20.3	17.6	**22.8**	**27.3**
Marital status				
Currently married/cohabiting	63.7	66.4	**61.1**	**62.6**
Divorced or separated/widowed	4.8	5.5	**4.3**	**4.1**
Never married	31.5	28.1	**34.6**	**33.3**
Religion of household				
Catholic	14.7	13.1	**15.8**	**17.6**
Other Christian	44.0	44.1	**45.4**	**44.7**
Muslim	39.2	41.2	**36.4**	**35.6**
Other	2.1	1.7	**2.4**	**2.0**
Ethnicity				
Hausa	21.0	22.8	**19.0**	**19.6**
Igbo	22.5	21.0	**23.6**	**24.6**
Other	56.5	56.2	**57.4**	**55.9**
Wealth				
Poorest	23.2	23.1	**22.2**	**20.2**
Second poorest	20.2	20.3	**20.2**	**20.3**
Middle	17.6	19.5	**16.4**	**15.4**
Second wealthiest	18.6	18.1	**19.4**	**19.3**
Wealthiest	20.5	19.1	**21.8**	**24.8**
Residence				
Rural	42.9	39.3	**44.7**	**46.0**
Urban	57.1	60.7	**55.3**	**54.0**
State				
Anambra	12.8	10.3	**14.4**	**15.4**
Kaduna	9.5	10.0	**8.9**	**7.9**
Kano	13.1	14.5	**11.2**	**12.5**
Lagos	21.4	22.4	**21.4**	**22.2**
Nasarawa	13.4	12.5	**14.3**	**12.8**
Rivers	17.0	17.8	**17.1**	**18.3**
Taraba	12.7	12.4	**12.6**	**10.9**
Parity				
0	35.1	31.9	**37.9**	35.8
1–2	25.1	24.8	**25.7**	25.0
3–4	21.7	23.2	**20.6**	21.3
5+	18.1	20.1	**15.8**	18.0
Current contraceptive use				
No	75.8	78.0	**73.5**	**71.4**
Yes	24.2	22.0	**26.5**	**28.6**
Unadjusted likely abortion incidence	41.1	30.3	**51.4**	39.4
Total	100.0	100.0	100.0	100.0

Estimates weighted; bold indicates *p* value for design-based *F* test (reference group 0 confidantes) less than 0.05

**Table 2:** Characteristics of female respondents age 15 to 49 overall and by number of female confidantes in Cote d’Ivoire

	All respondents	0 confidantes	≥ 1 confidante	≥ 2 confidantes
*N*	2738	959	1761	263
Age				
15–19	20.1	18.0	**21.1**	**21.5**
20–24	18.1	16.6	**19.0**	**23.5**
25–29	17.9	17.8	**17.8**	**14.9**
30–34	16.3	16.9	**16.0**	**14.2**
35–39	12.8	12.0	**13.3**	**19.3**
40–44	9.4	11.0	**8.5**	**4.5**
45–49	5.5	7.8	**4.3**	**2.1**
Education				
Never	45.2	50.3	**42.2**	40.1
Primary	25.9	26.2	**25.7**	28.0
Secondary	23.0	18.9	**25.2**	25.3
Higher	6.0	4.6	**6.8**	6.6
Marital status				
Currently married/cohabiting	64.8	68.8	**62.6**	**58.6**
Divorced or separated/widowed	4.4	4.4	**4.4**	**3.4**
Never married	30.8	26.8	**33.0**	**38.0**
Religion of household				
Muslim	39.5	38.8	39.8	35.5
Catholic	20.3	17.7	21.8	21.0
Evangelical	15.4	14.0	16.2	19.3
Other	13.7	14.8	13.1	15.8
No religion	11.1	14.7	9.0	8.4
Ethnicity				
Akan	34.6	36.8	33.5	36.2
Mande (north and south)	20.8	23.8	19.1	20.0
Gur	14.4	9.1	17.2	17.0
Other Ivoirian	9.3	8.7	9.7	10.9
Other non-Ivoirian	21.0	21.6	20.6	16.0
Wealth				
Poorest	20.1	22.4	18.7	19.9
Second poorest	20.0	19.3	20.5	23.5
Middle	17.1	17.5	16.9	14.1
Second wealthiest	19.7	22.0	18.3	18.8
Wealthiest	23.1	18.8	25.6	23.8
Residence				
Rural	38.5	40.3	37.7	40.2
Urban	61.5	59.7	62.3	59.8
Parity				
0	25.8	21.4	**28.0**	31.2
1–2	32.2	31.9	**32.4**	31.0
3–4	21.5	22.0	**21.2**	19.0
5+	20.6	24.7	**18.3**	18.8
Current contraceptive use				
No	75.0	77.7	73.3	70.6
Yes	25.0	22.3	26.7	29.4
Unadjusted likely abortion incidence	36.9	27.9	41.9	50.0
Total	100.0	100.0	100.0	100.0

Estimates weighted; bold indicates *p* value for design-based *F* test (reference group 0 confidantes) less than 0.05

**Table 3:** Characteristics of female respondents age 15 to 49 overall and by number of female confidantes in Rajasthan, India

	All respondents	0 confidantes	≥ 1 confidante	≥ 2 confidantes
*N*	5832	854	4912	1118
Age				
15–19	18.5	16.6	**18.9**	**20.9**
20–24	19.6	15.7	**20.5**	**23.6**
25–29	16.7	14.0	**17.2**	**17.3**
30–34	13.6	13.7	**13.6**	**14.1**
35–39	12.8	13.9	**12.5**	**11.3**
40–44	10.9	14.2	**10.2**	**8.2**
45–49	7.8	11.9	**7.0**	**4.7**
Education				
Never	36.8	47.9	**34.5**	**31.0**
Primary	24.0	22.9	**24.3**	**26.5**
Secondary	16.5	16.0	**16.6**	**14.5**
Higher	22.7	13.3	**24.6**	**28.0**
Marital status				
Currently married/cohabiting	76.4	80.5	**75.5**	**72.6**
Divorced or separated/widowed	2.6	3.4	**2.4**	**2.2**
Never married	21.0	16.1	**22.1**	**25.2**
Religion of household				
Hindu	85.9	79.9	**87.1**	80.6
Muslim	12.7	18.9	**11.5**	18.4
Other	1.4	1.3	**1.4**	1.0
Caste of household				
Scheduled caste	22.7	26.4	**21.8**	**29.1**
Scheduled tribe	11.7	8.6	**12.3**	**11.5**
Other backward caste	46.7	45.0	**47.1**	**44.7**
General	18.9	20.0	**18.7**	**14.8**
Wealth				
Poorest	16.0	24.2	**14.3**	**11.4**
Second poorest	17.8	16.1	**18.2**	**17.1**
Middle	20.1	15.3	**21.0**	**24.5**
Second wealthiest	22.8	25.1	**22.5**	**25.1**
Wealthiest	23.3	19.3	**24.0**	**21.9**
Residence				
Rural	65.4	62.9	65.8	**72.6**
Urban	34.6	37.1	34.2	**27.4**
Parity				
0	30.6	25.1	**31.9**	**33.9**
1–2	36.2	34.5	**36.4**	**35.9**
3–4	25.8	29.3	**25.1**	**22.8**
5+	7.4	11.1	**6.6**	**7.4**
Current contraceptive use				
No	51.8	54.1	51.6	54.7
Yes	48.2	45.9	48.4	45.3
Unadjusted likely abortion incidence	9.5	9.2	9.2	10.4
Total	100.0	100.0	100.0	100.0

Estimate weighted; bold indicates *p* value for design-based *F* test (reference group 0 confidantes) less than 0.05

### Assumption 1: transmission of abortion knowledge

Respondents reported on average 0.8 close confidantes in Nigeria, providing information on 5883 first and 1953 second confidantes; the corresponding numbers were 0.8, 1761, and 263 in Cote d’Ivoire and 1.1, 4921, and 1118 in Rajasthan. Forty-three percent of Nigerian respondents reported having no close confidantes while 35% and 15% of respondents reported having no close confidantes in Cote d’Ivoire and Rajasthan, respectively. Respondents in each country who reported having no confidantes tended to be older, less educated, and currently married or cohabiting compared to those with one or more confidantes (Tables 1, 2, and 3). There were additional differences in respondent characteristics among those with different numbers of reported confidantes by wealth in some countries and by country-specific variables like religion, caste, ethnicity, and state (Tables 1, 2, and 3). Women with no confidantes also tended to have more children and were less likely to be using contraception. We observed higher abortion incidence rates among respondents who reported at least one confidante in Nigeria and Cote d’Ivoire compared to those who reported none, although the difference is only statistically significant for respondents who reported at least one confidante in Nigeria; respondent abortion rates were similar by number of reported confidantes in Rajasthan (Tables 1, 2, and 3).

Among respondents who reported an abortion, the percentage who told a given confidante about the experience varied by context and confidante. In Nigeria, 51.1% of respondents reported an abortion and had at least one confidante indicated they told confidante one about the experience, while 32.8% who had a second confidante told her about that experience; in Cote d’Ivoire, 58.0% and 29.1% of respondents shared their abortion experience with confidantes one and two, respectively, while these numbers were 61.0% and 57.5% in Rajasthan (Table 4). Although these results provide evidence that direct transmission of respondent abortions via respondents telling confidantes is incomplete, we believe this suggests that the transmission of confidante abortions to respondents was similarly incomplete and thus Assumption 1 was likely violated. We provide additional details on abortion sharing by background characteristics elsewhere (see Additional file 1).

**Table 4:** Percentage of respondents who reported an abortion who shared it with each confidante

	Confidante 1		Confidante 2	
	%	*N*	%	*N*
Nigeria	51.1	175	32.8	50
Cote d’Ivoire	58.0	52	29.1	10
Rajasthan	61.0	51	57.5	17

### Assumption 2: confidante sample representativeness

Across settings, confidante one was on average significantly more educated than respondents, and confidante two was even more so (Table 5). In Rajasthan specifically, confidantes one and two were significantly younger than respondents (Table 5). Thus, Assumption 2 was violated.

**Table 5:** Characteristics of female respondents age 15 to 49 and their two closest female confidantes age 15 to 49 in Nigeria; Cote d’Ivoire; and Rajasthan, India

	Nigeria						Cote d’Ivoire						Rajasthan					
	Respondent		Confidante 1		Confidante 2		Respondent		Confidante 1		Confidante 2		Respondent		Confidante 1		Confidante 2	
	%	*N*	%	*N*	%	*N*	%	*N*	%	*N*	%	*N*	%	*N*	%	*N*	%	*N*
Mean age	29.1	11,106	28.4	5772	28.5	1923	28.5	2738	29.0	1756	27.5	262	29.1	5832	27.7	4911	26.5	1118
Age																		
15–19	18.9	2257	**19.0**	1163	**18.1**	382	20.1	542	17.9	305	22.4	56	18.5	1116	**20.0**	1035	**22.7**	276
20–24	16.2	1870	**19.6**	1132	**18.7**	352	18.1	500	17.9	307	20.9	52	19.6	1153	**22.3**	1071	**23.8**	264
25–29	18.8	2040	**18.0**	1073	**18.7**	381	17.9	495	16.0	298	16.4	45	16.7	986	**17.6**	870	**20.0**	212
30–34	15.0	1629	**15.3**	878	**17.4**	323	16.3	436	18.3	306	14.4	36	13.6	786	**14.0**	700	**14.3**	158
35–39	13.9	1473	**13.1**	694	**12.7**	230	12.8	351	13.6	255	14.0	41	12.8	738	**11.3**	523	**9.2**	107
40–44	10.5	1102	**9.3**	509	**9.6**	158	9.4	262	9.4	166	7.9	22	10.9	592	**8.6**	413	**4.9**	51
45–49	6.8	735	**5.7**	323	**4.9**	97	5.5	152	6.9	119	4.0	10	7.8	461	**6.2**	299	**5.2**	50
Education																		
Never	17.5	2355	**15.9**	1049	**16.1**	342	45.2	1254	**42.8**	773	**39.3**	110	36.8	2187	**32.3**	1626	**28.1**	291
Primary	15.2	1906	**11.3**	789	**8.2**	202	25.9	714	**20.7**	366	**19.6**	49	24.0	1400	**21.4**	1064	**20.8**	226
Secondary	46.9	4934	**46.4**	2687	**46.3**	894	23.0	615	**28.2**	484	**31.4**	80	16.5	938	**17.9**	888	**18.9**	223
Higher	20.3	1911	**26.3**	1345	**29.4**	508	6.0	152	**8.3**	134	**9.7**	23	22.7	1307	**28.4**	1334	**32.2**	378
Number of confidantes																		
0	45.1	4788	--	--	--	--	35.8	959	--	--	--	--	17.1	854	--	--	--	--
1	35.8	3930	--	--	--	--	54.3	1,498	--	--	--	--	65.2	3794	--	--	--	--
2+	19.1	1953	--	--	--	--	9.9	263	--	--	--	--	17.7	1118	--	--	--	--
Total	100.0	11,106	100.0	5883	100.0	1953	100.0	2738	100.0	1761	100.0	263	100.0	5832	100.0	4912	100.0	1118

Estimates weighted, Ns unweighted; bold indicates *p* value for design-based *F* test (reference respondents) less than 0.05

### Assumption 3: reduced social desirability pressure

The respondent 1-year incidence rate of abortion was 18.7 per 1000 women of reproductive age in Nigeria, 18.8 in Cote d’Ivoire, and 7.0 per 1000 in Rajasthan (Table 6). The unadjusted confidante one incidence was 48.0% higher than the respondent incidence in Nigeria, 24.5% higher in Cote d’Ivoire, and 44.6% higher in Rajasthan (Table 6). Unadjusted confidante two abortion incidence rates were 18.6% higher than respondent incidences in Nigeria, 76.4% higher in Cote d’Ivoire, and 57.6% higher in Rajasthan. None of these differences were statistically significant. As such, Assumption 3 was not met when using the unadjusted confidante data.

**Table 6:** One-year abortion incidence (per 1000) of female respondents age 15 to 49 and their closest female confidantes age 15 to 49 by country and adjustment for biases

	Respondent		Confidante 1^a^		Confidante 2^a^	
	Estimate	SE	Estimate	SE	Estimate	SE
**Nigeria**	*n* = 11,106		*n* = 5883		*n* = 1953	
Unadjusted	18.7	1.94	27.7	2.78	22.2	4.44
+ Less certain confidante abortions	--	--	**37.3**	3.63	**30.9**	5.88
+ Poisson adjustment for missing confidantes	--	--	**35.1**	2.04	**28.7**	1.65
**Cote d’Ivoire**	*n* = 2738		*n* = 1761		*n* = 263	
Unadjusted	18.8	3.56	23.4	4.14	33.2	11.35
+ Less certain confidante abortions	--	--	**32.3**	5.17	**42.0**	11.86
+ Poisson adjustment for missing confidantes	--	--	**31.5**	3.40	**46.3**	3.96
**Rajasthan**	*n* = 5832		*n* = 4912		*n* = 1118	
Unadjusted	7.0	1.24	10.2	3.91	11.1	4.30
+ Less certain confidante abortions	--	--	**15.6**	4.80	**17.1**	4.77
+ Poisson adjustment for missing confidantes	--	--	**15.2**	4.68	**16.7**	4.54

Bold indicates statistical significance in comparison to unadjusted respondent incidence

^a^Poisson modeled confidante estimates’ sample sizes are equivalent to the corresponding respondent sample size for that country

### Adjusting for violations of confidante method assumptions

To adjust for violations of Assumption 1 (incomplete transmission), we included confidante abortions that respondents reported with less certainty but where respondents still reported the method confidantes used. Compared to unadjusted estimates, this resulted in a 34.8% rise in confidante one abortion incidence in Nigeria, a 38.0% rise in Cote d’Ivoire, and a 53.5% rise in Rajasthan; the corresponding numbers for confidante two were 39.2%, 26.8%, and 54.7% (Table 6).

To adjust for selection bias (Assumption 2), we included the characteristics of respondents who reported zero confidantes in the confidante one sample and those who reported zero or one confidante in the confidante two sample and applied post-stratification weights to each sample. Results indicate that adjusted confidantes one and two age and education distributions were *not* statistically significantly different from that of the respondent in all countries after these adjustments, with the exception of Rajasthan confidante one education (although the distribution was qualitatively similar to that of the respondents). We present adjusted confidante age and education distributions elsewhere (see Additional files 2, 3, 4).

Incorporating the predicted probability of abortion for “missing” confidantes from the Poisson regression models—which adjusted for incomplete transmission (Assumption 1) and selection bias in the confidante samples (Assumption 2)—we found that both confidantes one and two estimates declined compared to the unadjusted confidante estimates, with the exception of Cote d’Ivoire confidante two. Compared to confidante one estimates adjusted only for transmission bias, the Poisson-adjusted confidante one abortion incidence was 5.9% lower in Nigeria, 2.6% lower in Cote d’Ivoire, and 2.4% lower in Rajasthan (Table 6). The corresponding percent changes for confidante two were 6.9% decrease in Nigeria, 10.2% increase in Cote d’Ivoire, and 2.5% decrease in Rajasthan. The Poisson models had high goodness-of-fit, with the chi-squared *p* values greater than 0.99 for all models except Rajasthan confidante two (*p* < 0.01).

Altogether, our adjustments to account for transmission bias and confidante sample selection bias resulted in significant changes to confidante one abortion estimates in each of the countries, increasing by 26.8%, 34.4%, and 49.8% in Nigeria, Cote d’Ivoire, and Rajasthan, respectively; corresponding confidante two abortion incidence changes were 29.5%, 39.7%, and 50.9% (Table 6). The final confidantes one and two 1-year abortion incidence estimates were statistically significantly higher than the corresponding respondent estimates at 35.1 and 28.7 in Nigeria, 31.5 and 46.3 in Cote d’Ivoire, and 15.2 and 16.7 in Rajasthan (Table 6).

## Discussion

Results from this study provide important insights into the performance of the confidante methodology in Nigeria, Cote d’Ivoire, and Rajasthan [28]. Findings suggest that this hybrid version of the ATPR and best friend approaches failed to meet the assumptions of the methodology before adjustment. However, we believe including the less certain respondent-reported confidante abortions—which increased the rates—at least partially counteracted for incomplete transmission (Assumption 1) while the Poisson model predicting the likelihood of abortion for the “missing” confidantes in conjunction with the post-stratification weights counteracted the confidante sample selection bias (Assumption 2)—which generally decreased the rates. Following these adjustments, the assumption of reduced social desirability pressure (Assumption 3) was also achieved as indicated by the consistently significantly higher confidante abortion incidence estimates compared to respondent estimates.

The extent to which the primary assumptions of the social network-based methodologies were met may partly explain why this methodology works differently according to social context. In India, almost all respondents had at least one confidante (85.4%), but only 61.0% and 57.5% directly shared their experience of abortion with their closest and next closest friends. In West Africa, fewer women reported a confidante (56.9 to 65.0%), and women were even less likely to share their abortion experience with a confidante (51.1% and 32.8% for confidantes one and two in Nigeria, 58.0% and 29.1% in Cote d’Ivoire), increasing the potential for both confidante sample distortion and transmission bias. However, this does not rule out the possibility that more women *know* about other women’s abortions in West Africa (regardless of whether they were directly *told* by the woman) because of greater reliance on one’s social network to access clandestine services. In contrast, because abortion is legal in Rajasthan, abortion procedures and drugs may be easier to access without input from one’s social network [24].

Altogether, the adjustments we made to account for transmission bias and confidante sample selection increased confidantes one and two abortion estimates by 26.8% and 29.5% in Nigeria, 34.4% and 39.7% in Cote d’Ivoire, and 49.8% and 50.9% in Rajasthan, respectively. The heterogeneity in these adjustments’ impact on the abortion incidence by country is substantial, suggesting the extent of assumption violations varies significantly by context. Evidence from the three countries involved in this study suggests that violations to Assumptions 1 and 2 were considerable, and only after adjustment was Assumption 3 true. More research is needed to better understand how and when women share their abortion experiences with friends and the extent of methodological biases in individual countries in order to determine the appropriateness of using this method in other contexts.

Based on these results and the associated incidence estimates produced after making the aforementioned adjustments, we believe the confidante methodology performed better in Western African contexts where abortion is legally restricted and women may need to consult more people (not necessarily a close female confidante) to navigate accessing care. However, this methodology does not eliminate concerns of continued bias, particularly in Rajasthan where the indirect estimate is still lower than one might expect based on available evidence [3]. In this context, abortion is legal and may be more readily available, not requiring women to draw on their social networks’ knowledge [24]. The confidante approach may have also been less effective at overcoming social desirability bias in Rajasthan as abortion, while legal, remains a contentious public issue due to mass-media, regulatory, and punitive actions taken by the government to curb the practice of sex-selective abortions on account of son preference and the resulting skewed sex ratios [38]. Additionally, although knowledge of friends’ abortions may still be incomplete in Rajasthan, we did not observe a decline in abortion sharing between confidantes one and two the way we did in Nigeria and Cote d’Ivoire. This finding suggests that in some settings, using only one confidante would result in more accurate abortion estimates as women appear more likely to know about their *closest* female confidantes’ abortions. The greater distortion in confidante two characteristics compared to respondents further supports use of only one confidante.

Comparing our Nigerian (35.1) and Ivorian (31.5) results to available regional estimates based on Bayesian modeling illustrates our results are similar; the West Africa 1-year abortion incidence was 31 per 1000 [1]. Our Nigerian abortion rate was only minimally higher than the most recent country-specific national estimate of 33 abortions per 1000 women age 15 to 49 obtained using the AICM methodology in 2012 [39]. With increasing availability of medication abortion drugs and declining desired fertility in Nigeria in 7 years since the AICM data collection, one might expect the rate of abortion to have increased in this setting. No abortion incidence estimates are available in Cote d’Ivoire to make such a comparison. In the Indian context, our abortion estimates are lower than the national incidence of 47 abortions per 1000 recently published for the country [3]. Our lower estimate may signal the poor performance of social network-based indirect methodologies in the Indian context, which has been suggested in previous studies [25], but may also reflect differences in reproductive health indicators in Rajasthan compared to India as a whole, as the contraceptive prevalence rate is higher than national estimates [40] and the distribution of mifepristone and misoprostol combination packs are lower compared to other states [41–43]. In particular, the Rajasthan government has conducted raids of pharmacies and chemists in recent years, and other research suggests that fear of legal repercussions or fines has led some outlets to stop distributing medication abortion drugs altogether [42]. This indicates we might expect a lower abortion rate in Rajasthan than other states. The extent to which our confidante rate may still be an underestimate is unknown given we lack an external, objective measure against which to validate our findings.

While our more descriptive wording of abortion may have captured more abortion experiences than questions including direct translations of “induced abortion,” we do not think we have captured all of the abortion experiences or post-coital behaviors women use to try to control their fertility in these settings. In this study, we also collected data on women’s experience doing something to “bring back their period at a time when they were worried they were pregnant;” however, exploration of this alternative question wording and the impact on incidence estimates is beyond the scope of this article.

This study has a number of limitations. While the Poisson regression addresses some of the issues of confidante sampling and associated selection biases, there is the potential for unobserved factors that may distort the estimation of abortion rates among the confidante samples. We have limited information about the characteristics of the confidantes and the respondent’s pattern of communication with the confidante (we do not know when the respondent last communicated with the confidante and made the assumption that confidantes would have shared a recent abortion with the respondents). Additionally, defining confidantes as only those with whom the respondent reciprocally shared personal information may have biased the confidante estimates upwards. The fact that a significant proportion of women reported no such relation in Nigeria and Cote d’Ivoire suggests the narrow definition may have been problematic; researchers had similar concerns with regard to the ATPR’s implementation in Burkina Faso [24]. There is also a possibility of more than one respondent reporting the same woman as their closest or second closest confidante. Given we were selecting 35 to 40 households from each EA of 200 or more households and that confidantes do not have to reside in respondent’s community, we think the likelihood of double counting confidantes is unlikely. However, double counting would not significantly bias our results since any double counting would apply to both the numerator and the denominator [2]. With regard to transmission bias, our means of assessing the visibility of abortions in these communities was to ascertain whether respondents who reported their own abortion “told” specific individuals, including each of her confidantes. However, in asking about the confidantes’ abortions, we did not ask for only those about which the confidante had “told” the respondent. Future work may better capture the visibility of abortions between friends by simply asking respondents if it is likely that a confidante *knows* about her abortion [44]. Assessing the methods’ performance and the appropriateness of adjustments for transmission bias through use of related but less stigmatizing outcomes, like long-acting reversible contraceptive use, may offer a suitable confidante methodology validation test in future implementations [44]. Lastly, some women may have mistakenly reported spontaneous abortions in response to the questions on pregnancy removal, which would bias our estimates upward.

Despite the aforementioned limitations, this study has a number of strengths. Samples are large and diverse, and contexts vary with regard to abortion legality. Investigators collected data contemporaneously and employed the same piloting, training, and data collection methodologies, providing a robust assessment of the performance of this methodology. Asking general abortion questions and about the confidantes’ experiences with abortion prior to asking the respondents about their own experience may have improved self-reported data. Additionally, the analytic approach adjusts for potential assumption violations in the confidante abortion incidence estimates as previously discussed.

## Conclusion

Many countries currently have limited knowledge about the extent of induced abortion locally, the demography of women who terminate a pregnancy, and risk-factors for abortion-related morbidity and mortality. Current results suggest that the confidante approach, which enables the collection of confidante characteristics and abortion details, may present an opportunity to address some abortion-related data deficiencies, particularly in legally restrictive settings. However, further research is needed to determine a priori in which contexts social network-based methods, like the confidante methodology, perform best. Additionally, more research is required on transmission bias and confidante relationship criteria. Depending on the research objectives and the size of the respondent sample, collecting data on respondents’ single closest confidante may be sufficient and may result in less biased data than that of a second or higher order confidante. Future studies using this approach could benefit from collecting additional information on the confidante(s), which could help to generate weights and models that better account for confidante selection bias. Subsequent work can also explore alternative weighting approaches to account for the observed sources of bias to produce a singular estimate of abortion for a given context that perhaps more effectively incorporates data from respondents and higher order confidantes. Lastly, using question wording that captures a broader range of post-coital behaviors to regulate one’s fertility warrants further exploration. More broadly, with further refinements of the methodology and associated adjustments for biases, there is potential for researchers to use this social network-based approach to study other stigmatized outcomes and improve our understanding of many clandestine behaviors.

## Supplementary information

**Additional file 1. **Among respondents who reported an abortion, percentage who shared it with each of their confidantes, overall and by background characteristics. Estimates weighted; bold indicates *p*-value for design-based F test less than 0.05.

**Additional file 2. **Characteristics of female respondents age 15 to 49 and their two closest female confidantes age 15 to 49 in Nigeria. Estimates weighted, Ns unweighted; bold indicates *p*-value for design-based F test (reference respondents) less than 0.05. ^2^Estimate include respondent characteristics in place of "missing" confidantes; applied post-stratification weights.

**Additional file 3. **Characteristics of female respondents age 15 to 49 and their two closest female confidantes age 15 to 49 in Cote d'Ivoire. Estimates weighted, Ns unweighted; bold indicates *p*-value for design-based F test (reference respondents) less than 0.05. ^2^Estimate include respondent characteristics in place of "missing" confidantes.

**Additional file 4. **Characteristics of female respondents age 15 to 49 and their two closest female confidantes age 15 to 49 in Rajasthan, India. Estimates weighted, Ns unweighted; bold indicates *p*-value for design-based F test (reference respondents) less than 0.05. Estimate include respondent characteristics in place of "missing" confidantes; post-stratification weights applied.

## Data Availability

The datasets analyzed during the current study are available in the Performance Monitoring for Action (formerly Performance Monitoring and Accountability 2020) repository, available upon request at www.pmadata.org.
