# Supplementary material for: Social network-based measurement of abortion incidence: promising findings from population-based surveys in Nigeria, Cote d’Ivoire, and Rajasthan, India
Source: Popul Health Metr. 2020 Oct 19;18:28. doi: 10.1186/s12963-020-00235-y (PMC7574299; doi:10.1186/s12963-020-00235-y)
Supplement: Supplementary file 1 — Additional file 1. Among respondents who reported an abortion, percentage who shared it with each of their confidantes, overall and by background characteristics. Estimates weighted; bold indicates p-value for design-based F test less than 0.05. [file 12963_2020_235_MOESM1_ESM.docx]

**Additional file 1. Among respondents who reported an abortion, percentage who shared it with each of their confidantes, overall and by background characteristics^1^**

|  |  | Nigeria | | | |  | Cote d'Ivoire | | | |  | Rajasthan | | | |
| --- | --- | --- | --- | --- | --- | --- | --- | --- | --- | --- | --- | --- | --- | --- | --- |
|  |  | Confidante 1 | | Confidante 2 | |  | Confidante 1 | | Confidante 2 | |  | Confidante 1 | | Confidante 2 | |
|  |  | % | N | % | N |  | % | N | % | N |  | % | N | % | N |
| Age | |  |  |  |  |  |  |  |  |  |  |  |  |  |  |
|  | 15-19 | 62.2 | 25 | 64.8 | 5 |  | 52.2 | 6 | 0.0 | 1 |  | N/A | 0 | N/A | 0 |
|  | 20-24 | 66.0 | 46 | 23.2 | 12 |  | 62.3 | 18 | 46.8 | 2 |  | 53.0 | 21 | 44.4 | 5 |
|  | 25-29 | 54.8 | 37 | 28.1 | 11 |  | 76.4 | 11 | 70.7 | 2 |  | 70.2 | 17 | 70.2 | 2 |
|  | 30-34 | 34.5 | 32 | 36.3 | 10 |  | 48.3 | 10 | 32.1 | 2 |  | 13.9 | 4 | 13.9 | 2 |
|  | 35-39 | 39.8 | 21 | 43.6 | 7 |  | 39.5 | 7 | 0.0 | 3 |  | 100.0 | 7 | 100.0 | 1 |
|  | 40-44 | 26.9 | 10 | 3.8 | 3 |  | N/A | 0 | N/A | 0 |  | N/A | 0 | N/A | 0 |
|  | 45-49 | 31.9 | 4 | 60.3 | 2 |  | N/A | 0 | N/A | 0 |  | N/A | 0 | N/A | 0 |
| Education | |  |  |  |  |  |  |  |  |  |  |  |  |  |  |
|  | Never | 44.7 | 10 | **33.7** | 4 |  | 42.2 | 15 | 29.1 | 5 |  | 58.7 | 11 | 58.7 | 4 |
|  | Primary | 38.6 | 25 | **76.6** | 6 |  | 56.0 | 12 | 0.0 | 2 |  | 57.8 | 19 | 57.8 | 6 |
|  | Secondary | 31.7 | 101 | **15.6** | 27 |  | 80.8 | 17 | 100.0 | 1 |  | 45.6 | 10 | 28.8 | 3 |
|  | Higher | 40.4 | 39 | **49.8** | 13 |  | 39.5 | 8 | 0.0 | 2 |  | 92.7 | 11 | 92.7 | 4 |
| Marital status | |  |  |  |  |  |  |  |  |  |  |  |  |  |  |
|  | Currently married/cohabiting | **40.6** | 95 | **14.5** | 28 |  | 46.9 | 27 | 30.6 | 6 |  | 60.2 | 50 | 56.7 | 16 |
|  | Divorced or separated/widowed | **78.6** | 10 | **100.0** | 4 |  | 63.5 | 3 | 100.0 | 1 |  | N/A | 0 | N/A | 0 |
|  | Never married | **60.0** | 70 | **48.1** | 18 |  | 75.0 | 22 | 0.0 | 3 |  | 100.0 | 4 | 100.0 | 1 |
| Wealth | |  |  |  |  |  |  |  |  |  |  |  |  |  |  |
|  | Poorest | 49.5 | 24 | 34.2 | 4 |  | **21.5** | 9 | 0.0 | 1 |  | 0.0 | 8 | **0.0** | 3 |
|  | Second poorest | 60.0 | 32 | 77.7 | 8 |  | **66.2** | 7 | 32.1 | 2 |  | 77.5 | 11 | **77.5** | 2 |
|  | Middle | 46.0 | 36 | 27.7 | 12 |  | **64.7** | 4 | 0.0 | 1 |  | 77.3 | 12 | **77.3** | 6 |
|  | Second wealthiest | 44.1 | 45 | 20.1 | 13 |  | **73.5** | 17 | 38.7 | 5 |  | 100.0 | 9 | **0.0** | 1 |
|  | Wealthiest | 57.7 | 38 | 30.1 | 13 |  | **56.7** | 15 | 0.0 | 1 |  | 85.7 | 11 | **85.7** | 5 |
| Residence | |  |  |  |  |  |  |  |  |  |  |  |  |  |  |
|  | Rural | 54.8 | 65 | 34.2 | 17 |  | 46.6 | 17 | 26.5 | 3 |  | 53.4 | 37 | 49.2 | 15 |
|  | Urban | 49.5 | 110 | 32.4 | 33 |  | 62.7 | 35 | 30.0 | 7 |  | 100.0 | 14 | 100.0 | 2 |
| Agrees woman who has abortion brings shame to family | | | |  |  |  |  |  |  |  |  |  |  |  |  |
|  | Yes | 54.7 | 69 | 29.8 | 21 |  | 54.9 | 31 | 17.3 | 7 |  | 41.1 | 19 | 41.1 | 5 |
|  | No | 47.5 | 106 | 36.0 | 29 |  | 62.6 | 21 | 54.1 | 3 |  | 67.6 | 32 | 63.0 | 12 |
| Agrees women who has abortion should not tell anyone | | | |  |  |  |  |  |  |  |  |  |  |  |  |
|  | Yes | 52.2 | 93 | 27.2 | 32 |  | **69.0** | 27 | 30.8 | 3 |  | 57.4 | 27 | 49.1 | 7 |
|  | No | 49.6 | 82 | 42.4 | 18 |  | **47.4** | 25 | 28.2 | 7 |  | 63.5 | 24 | 63.5 | 10 |
| Total | | 51.1 | 175 | 32.8 | 50 |  | 58.0 | 52 | 29.1 | 10 |  | 61.0 | 51 | 57.5 | 17 |

**^1^**Estimates weighted; bold indicates p-value for design-based F test less than 0.05

|  |  | Nigeria | | | |  | Cote d'Ivoire | | | |  | Rajasthan | | | |
| --- | --- | --- | --- | --- | --- | --- | --- | --- | --- | --- | --- | --- | --- | --- | --- |
|  |  | Confidante 1 | | Confidante 2 | |  | Confidante 1 | | Confidante 2 | |  | Confidante 1 | | Confidante 2 | |
|  |  | % | N | % | N |  | % | N | % | N |  | % | N | % | N |
| Age | |  |  |  |  |  |  |  |  |  |  |  |  |  |  |
|  | 15-19 | 62.2 | 25 | 64.8 | 5 |  | 52.2 | 6 | 0.0 | 1 |  | N/A | 0 | N/A | 0 |
|  | 20-24 | 66.0 | 46 | 23.2 | 12 |  | 62.3 | 18 | 46.8 | 2 |  | 53.0 | 21 | 44.4 | 5 |
|  | 25-29 | 54.8 | 37 | 28.1 | 11 |  | 76.4 | 11 | 70.7 | 2 |  | 70.2 | 17 | 70.2 | 2 |
|  | 30-34 | 34.5 | 32 | 36.3 | 10 |  | 48.3 | 10 | 32.1 | 2 |  | 13.9 | 4 | 13.9 | 2 |
|  | 35-39 | 39.8 | 21 | 43.6 | 7 |  | 39.5 | 7 | 0.0 | 3 |  | 100.0 | 7 | 100.0 | 1 |
|  | 40-44 | 26.9 | 10 | 3.8 | 3 |  | N/A | 0 | N/A | 0 |  | N/A | 0 | N/A | 0 |
|  | 45-49 | 31.9 | 4 | 60.3 | 2 |  | N/A | 0 | N/A | 0 |  | N/A | 0 | N/A | 0 |
| Education | |  |  |  |  |  |  |  |  |  |  |  |  |  |  |
|  | Never | 44.7 | 10 | **33.7** | 4 |  | 42.2 | 15 | 29.1 | 5 |  | 58.7 | 11 | 58.7 | 4 |
|  | Primary | 38.6 | 25 | **76.6** | 6 |  | 56.0 | 12 | 0.0 | 2 |  | 57.8 | 19 | 57.8 | 6 |
|  | Secondary | 31.7 | 101 | **15.6** | 27 |  | 80.8 | 17 | 100.0 | 1 |  | 45.6 | 10 | 28.8 | 3 |
|  | Higher | 40.4 | 39 | **49.8** | 13 |  | 39.5 | 8 | 0.0 | 2 |  | 92.7 | 11 | 92.7 | 4 |
| Marital status | |  |  |  |  |  |  |  |  |  |  |  |  |  |  |
|  | Currently married/cohabiting | **40.6** | 95 | **14.5** | 28 |  | 46.9 | 27 | 30.6 | 6 |  | 60.2 | 50 | 56.7 | 16 |
|  | Divorced or separated/widowed | **78.6** | 10 | **100.0** | 4 |  | 63.5 | 3 | 100.0 | 1 |  | N/A | 0 | N/A | 0 |
|  | Never married | **60.0** | 70 | **48.1** | 18 |  | 75.0 | 22 | 0.0 | 3 |  | 100.0 | 4 | 100.0 | 1 |
| Wealth | |  |  |  |  |  |  |  |  |  |  |  |  |  |  |
|  | Poorest | 49.5 | 24 | 34.2 | 4 |  | **21.5** | 9 | 0.0 | 1 |  | 0.0 | 8 | **0.0** | 3 |
|  | Second poorest | 60.0 | 32 | 77.7 | 8 |  | **66.2** | 7 | 32.1 | 2 |  | 77.5 | 11 | **77.5** | 2 |
|  | Middle | 46.0 | 36 | 27.7 | 12 |  | **64.7** | 4 | 0.0 | 1 |  | 77.3 | 12 | **77.3** | 6 |
|  | Second wealthiest | 44.1 | 45 | 20.1 | 13 |  | **73.5** | 17 | 38.7 | 5 |  | 100.0 | 9 | **0.0** | 1 |
|  | Wealthiest | 57.7 | 38 | 30.1 | 13 |  | **56.7** | 15 | 0.0 | 1 |  | 85.7 | 11 | **85.7** | 5 |
| Residence | |  |  |  |  |  |  |  |  |  |  |  |  |  |  |
|  | Rural | 54.8 | 65 | 34.2 | 17 |  | 46.6 | 17 | 26.5 | 3 |  | 53.4 | 37 | 49.2 | 15 |
|  | Urban | 49.5 | 110 | 32.4 | 33 |  | 62.7 | 35 | 30.0 | 7 |  | 100.0 | 14 | 100.0 | 2 |
| Agrees woman who has abortion brings shame to family | | | |  |  |  |  |  |  |  |  |  |  |  |  |
|  | Yes | 54.7 | 69 | 29.8 | 21 |  | 54.9 | 31 | 17.3 | 7 |  | 41.1 | 19 | 41.1 | 5 |
|  | No | 47.5 | 106 | 36.0 | 29 |  | 62.6 | 21 | 54.1 | 3 |  | 67.6 | 32 | 63.0 | 12 |
| Agrees women who has abortion should not tell anyone | | | |  |  |  |  |  |  |  |  |  |  |  |  |
|  | Yes | 52.2 | 93 | 27.2 | 32 |  | **69.0** | 27 | 30.8 | 3 |  | 57.4 | 27 | 49.1 | 7 |
|  | No | 49.6 | 82 | 42.4 | 18 |  | **47.4** | 25 | 28.2 | 7 |  | 63.5 | 24 | 63.5 | 10 |
| Total | | 51.1 | 175 | 32.8 | 50 |  | 58.0 | 52 | 29.1 | 10 |  | 61.0 | 51 | 57.5 | 17 |
| **^1^**Estimates weighted; bold indicates p-value for design-based F test less than 0.05 | | | | | | | |  |  |  |  |  |  |  |  |
