# Supplementary material for: Social network-based measurement of abortion incidence: promising findings from population-based surveys in Nigeria, Cote d’Ivoire, and Rajasthan, India
Source: Popul Health Metr. 2020 Oct 19;18:28. doi: 10.1186/s12963-020-00235-y (PMC7574299; doi:10.1186/s12963-020-00235-y)
Supplement: Supplementary file 2 — Additional file 2. Characteristics of female respondents age 15 to 49 and their two closest female confidantes age 15 to 49 in Nigeria. Estimates weighted, Ns unweighted; bold indicates p-value for design-based F test (reference respondents) less than 0.05. 2Estimate include respondent characteristics in place of "missing" confidantes; applied post-stratification weights. [file 12963_2020_235_MOESM2_ESM.docx]

**Additional file 2. Characteristics of female respondents age 15 to 49 and their two closest female confidantes age 15 to 49 in Nigeria^1^**

|  |  | Respondent | | Unadjusted Confidante 1 | | Adjusted Confidante 1^2^ | | Unadjusted Confidante 2 | | Adjusted Confidante 2^2^ | |
| --- | --- | --- | --- | --- | --- | --- | --- | --- | --- | --- | --- |
|  |  | % | N | % | N | % | N | % | N | % | N |
| Mean age | | 29.1 | 11,106 | 28.4 | 5,772 | 29.1 | 11,106 | 28.5 | 1,923 | 29.0 | 11,106 |
| Age | |  |  |  |  |  |  |  |  |  |  |
|  | 15-19 | 18.9 | 2,257 | **19.0** | 1,163 | 18.5 | 2,221 | **18.1** | 382 | 18.7 | 2,262 |
|  | 20-24 | 16.2 | 1,870 | **19.6** | 1,132 | 16.9 | 1,942 | **18.7** | 352 | 16.5 | 1,903 |
|  | 25-29 | 18.8 | 2,040 | **18.0** | 1,073 | 18.0 | 2,008 | **18.7** | 381 | 18.7 | 2,048 |
|  | 30-34 | 15.0 | 1,629 | **15.3** | 878 | 15.0 | 1,658 | **17.4** | 323 | 15.3 | 1,650 |
|  | 35-39 | 13.9 | 1,473 | **13.1** | 694 | 14.3 | 1,447 | **12.7** | 230 | 13.8 | 1,440 |
|  | 40-44 | 10.5 | 1,102 | **9.3** | 509 | 10.5 | 1,114 | **9.6** | 158 | 10.4 | 1,088 |
|  | 45-49 | 6.8 | 735 | **5.7** | 323 | 6.9 | 716 | **4.9** | 97 | 6.6 | 715 |
| Education | |  |  |  |  |  |  |  |  |  |  |
|  | Never | 17.5 | 2355 | **15.9** | 1,049 | 17.9 | 2,406 | **16.1** | 342 | 17.5 | 2,369 |
|  | Primary | 15.2 | 1,906 | **11.3** | 789 | 14.4 | 1,742 | **8.2** | 202 | 14.8 | 1,828 |
|  | Secondary | 46.9 | 4934 | **46.4** | 2,687 | 46.3 | 4,883 | **46.3** | 894 | 47.2 | 4,964 |
|  | Higher | 20.3 | 1911 | **26.3** | 1,345 | 21.4 | 2,075 | **29.4** | 508 | 20.5 | 1,945 |
| Number of confidantes | |  |  |  |  |  |  |  |  |  |  |
|  | 0 | 45.1 | 4,788 | -- | -- | -- | -- | -- | -- | -- | -- |
|  | 1 | 35.8 | 3,930 | -- | -- | -- | -- | -- | -- | -- | -- |
|  | 2+ | 19.1 | 1,953 | -- | -- | -- | -- | -- | -- | -- | -- |
| Total | | 100.0 | 11,106 | 100.0 | 5,883 | 100.0 | 11,106 | 100.0 | 1,953 | 100.0 | 11,106 |

**^1^**Estimates weighted, Ns unweighted; bold indicates p-value for design-based F test (reference respondents) less than 0.05

^2^Estimates include respondent characteristics in place of "missing" confidantes; post-stratification weights applied
