# Supplementary material for: Social network-based measurement of abortion incidence: promising findings from population-based surveys in Nigeria, Cote d’Ivoire, and Rajasthan, India
Source: Popul Health Metr. 2020 Oct 19;18:28. doi: 10.1186/s12963-020-00235-y (PMC7574299; doi:10.1186/s12963-020-00235-y)
Supplement: Supplementary file 3 — Additional file 3. Characteristics of female respondents age 15 to 49 and their two closest female confidantes age 15 to 49 in Cote d'Ivoire. Estimates weighted, Ns unweighted; bold indicates p-value for design-based F test (reference respondents) less than 0.05. 2Estimate include respondent characteristics in place of "missing" confidantes. [file 12963_2020_235_MOESM3_ESM.docx]

**Additional file 3. Characteristics of female respondents age 15 to 49 and their two closest female confidantes age 15 to 49 in Cote d'Ivoire^1^**

|  |  | Respondent | | Unadjusted Confidante 1 | | Adjusted Confidante 1^2^ | | Unadjusted Confidante 2 | | Adjusted Confidante 2^2^ | |
| --- | --- | --- | --- | --- | --- | --- | --- | --- | --- | --- | --- |
|  |  | % | N | % | N | % | N | % | N | % | N |
| Mean age | | 28.5 | 2,738 | 29.0 | 1,756 | 28.8 | 2,738 | 27.5 | 262 | 28.5 | 2,738 |
| Age | |  |  |  |  |  |  |  |  |  |  |
|  | 15-19 | 20.1 | 542 | 17.9 | 305 | 19.0 | 484 | 22.4 | 56 | 20.1 | 542 |
|  | 20-24 | 18.1 | 500 | 17.9 | 307 | 17.8 | 481 | 20.9 | 52 | 17.9 | 496 |
|  | 25-29 | 17.9 | 495 | 16.0 | 298 | 17.2 | 470 | 16.4 | 45 | 18.0 | 498 |
|  | 30-34 | 16.3 | 436 | 18.3 | 306 | 17.2 | 462 | 14.4 | 36 | 16.3 | 433 |
|  | 35-39 | 12.8 | 351 | 13.6 | 255 | 12.7 | 370 | 14.0 | 41 | 12.4 | 344 |
|  | 40-44 | 9.4 | 262 | 9.4 | 166 | 9.7 | 275 | 7.9 | 22 | 9.6 | 269 |
|  | 45-49 | 5.5 | 152 | 6.9 | 119 | 6.4 | 196 | 4.0 | 10 | 5.7 | 156 |
| Education | |  |  |  |  |  |  |  |  |  |  |
|  | Never | 45.2 | 1,254 | **42.8** | 773 | 45.3 | 1,267 | **39.3** | 110 | 45.1 | 1,251 |
|  | Primary | 25.9 | 714 | **20.7** | 366 | 24.4 | 621 | **19.6** | 49 | 25.5 | 691 |
|  | Secondary | 23.0 | 615 | **28.2** | 484 | 23.9 | 672 | **31.4** | 80 | 23.2 | 634 |
|  | Higher | 6.0 | 152 | **8.3** | 134 | 6.5 | 176 | **9.7** | 23 | 6.2 | 159 |
| Number of confidantes | |  |  |  |  |  |  |  |  |  |  |
|  | 0 | 35.8 | 959 | -- | -- | -- | -- | -- | -- | -- | -- |
|  | 1 | 54.3 | 1,498 | -- | -- | -- | -- | -- | -- | -- | -- |
|  | 2+ | 9.9 | 263 | -- | -- | -- | -- | -- | -- | -- | -- |
| Total | | 100.0 | 2,738 | 100.0 | 1,761 | 100.0 | 2,738 | 100.0 | 263 | 100.0 | 2,738 |

**^1^**Estimates weighted, Ns unweighted; bold indicates p-value for design-based F test (reference respondents) less than 0.05

^2^Estimates include respondent characteristics in place of "missing" confidantes; post-stratification weights applied
