# Supplementary material for: Social network-based measurement of abortion incidence: promising findings from population-based surveys in Nigeria, Cote d’Ivoire, and Rajasthan, India
Source: Popul Health Metr. 2020 Oct 19;18:28. doi: 10.1186/s12963-020-00235-y (PMC7574299; doi:10.1186/s12963-020-00235-y)
Supplement: Supplementary file 4 — Additional file 4. Characteristics of female respondents age 15 to 49 and their two closest female confidantes age 15 to 49 in Rajasthan, India. Estimates weighted, Ns unweighted; bold indicates p-value for design-based F test (reference respondents) less than 0.05. Estimate include respondent characteristics in place of "missing" confidantes; post-stratification weights applied. [file 12963_2020_235_MOESM4_ESM.docx]

**Additional file 4. Characteristics of female respondents age 15 to 49 and their two closest female confidantes age 15 to 49 in Rajasthan, India^1^**

|  |  | Respondent | | Unadjusted Confidante 1 | | Adjusted Confidante 1^2^ | | Unadjusted Confidante 2 | | Adjusted Confidante 2^2^ | |
| --- | --- | --- | --- | --- | --- | --- | --- | --- | --- | --- | --- |
|  |  | % | N | % | N | % | N | % | N | % | N |
| Mean age | | 29.1 | 5,832 | 27.7 | 4,911 | 28.3 | 5,832 | 26.5 | 1,118 | 28.7 | 5,832 |
| Age | |  |  |  |  |  |  |  |  |  |  |
|  | 15-19 | 18.5 | 1,116 | **20.0** | 1,035 | 19.1 | 1,186 | **22.7** | 276 | 18.4 | 1,144 |
|  | 20-24 | 19.6 | 1,153 | **22.3** | 1,071 | 21.1 | 1,216 | **23.8** | 264 | 19.7 | 1,146 |
|  | 25-29 | 16.7 | 986 | **17.6** | 870 | 17.2 | 1,004 | **20.0** | 212 | 18.0 | 1,001 |
|  | 30-34 | 13.6 | 786 | **14.0** | 700 | 13.6 | 823 | **14.3** | 158 | 14.1 | 799 |
|  | 35-39 | 12.8 | 738 | **11.3** | 523 | 12.4 | 655 | **9.2** | 107 | 11.6 | 721 |
|  | 40-44 | 10.9 | 592 | **8.6** | 413 | 9.5 | 539 | **4.9** | 51 | 9.8 | 564 |
|  | 45-49 | 7.8 | 461 | **6.2** | 299 | 7.2 | 409 | **5.2** | 50 | 8.5 | 457 |
| Education | |  |  |  |  |  |  |  |  |  |  |
|  | Never | 36.8 | 2,187 | **32.3** | 1,626 | **34.8** | 2,065 | **28.1** | 291 | 38.0 | 2,155 |
|  | Primary | 24.0 | 1400 | **21.4** | 1,064 | **22.7** | 1,275 | **20.8** | 226 | 23.7 | 1,339 |
|  | Secondary | 16.5 | 938 | **17.9** | 888 | **16.8** | 1,031 | **18.9** | 223 | 15.4 | 987 |
|  | Higher | 22.7 | 1307 | **28.4** | 1,334 | **25.7** | 1,461 | **32.2** | 378 | 22.9 | 1,351 |
| Number of confidantes | |  |  |  |  |  |  |  |  |  |  |
|  | 0 | 17.1 | 854 | -- | -- | -- | -- | -- | -- | -- | -- |
|  | 1 | 65.2 | 3,794 | -- | -- | -- | -- | -- | -- | -- | -- |
|  | 2+ | 17.7 | 1,118 | -- | -- | -- | -- | -- | -- | -- | -- |
| Total | | 100.0 | 5,832 | 100.0 | 4,912 | 100.0 | 5,832 | 100.0 | 1,118 | 100.0 | 5,832 |

**^1^**Estimates weighted, Ns unweighted; bold indicates p-value for design-based F test (reference respondents) less than 0.05

^2^Estimates include respondent characteristics in place of "missing" confidantes; post-stratification weights applied
